# Prevalence and Determinants of Mucous Membrane Irritations in a Community Near a Cement Factory in Zambia: A Cross Sectional Study

**DOI:** 10.3390/ijerph120100871

**Published:** 2015-01-16

**Authors:** Emmy Nkhama, Micky Ndhlovu, J. Timothy Dvonch, Seter Siziya, Kuku Voyi

**Affiliations:** 1 Department of Environmental Health/Clinical Medicine, Chainama College of Health Sciences, P.O. Box 33991, Lusaka 10101, Zambia; E-Mail: makobani@yahoo.com; 2 Department of Environmental Health Sciences, School of Public Health, University of Michigan, Ann Arbor, MI 48109, USA; E-Mail: dvonch@umich.edu; 3 School of Public Health and Health System, Health Sciences Faculty, University of Pretoria, P.O. Box 667, Pretoria 0001, South Africa; E-Mail: kuku.voyi@gmail.com; 4 School of Medicine, Public Health Unit, Copperbelt University, P.O. Box 71191, Ndola 10101, Zambia; E-Mail: ssiziya@gmail.com; 5 School of Health Sciences, University of Lusaka, P.O. Box 36711, Lusaka 10101, Zambia

**Keywords:** cement production emissions, air pollution, mucous membrane, community, Zambia

## Abstract

Exposure to cement dust has been associated with deleterious health effects in humans. This study investigated whether residing near a cement factory increases the risk of irritations to the mucous membranes of the eyes and respiratory system. A cross sectional study was conducted in Freedom Compound, a community bordering a cement factory in Chilanga, Zambia and a control community, Bauleni, located 18 km from the cement plant. A modified American Thoracic Society questionnaire was administered to 225 and 198 respondents aged 15–59 years from Freedom and Bauleni, respectively, to capture symptoms of the irritations. Respondents from Freedom Compound, were more likely to experience the irritations; adjusted ORs 2.50 (95% CI: 1.65, 3.79), 4.36 (95% CI (2.96, 6.55)) and 1.94 (95% CI (1.19, 3.18)) for eye, nose and sinus membrane irritations respectively. Cohort panel studies to determine associations of cement emissions to mucous membrane irritations and respiratory symptoms, coupled with field characterization of the exposure are needed to assess whether the excess prevalence of symptoms of mucous membrane irritations observed in Freedom compound are due to emissions from the cement factory.

## 1. Introduction

Cement production inevitably leads to environmental pollution. Emissions from cement production plants include carbon monoxide (CO), carbon dioxide (CO_2_), nitrous oxides (NOx), sulphur oxides (SOx) and particulate matter (PM). Other emissions include dioxins and furans (PCDD/FS), polycyclic aromatic hydrocarbons (PAHs), poly chlorinated biphenyls (PCBs), benzene and other organic compounds. These emissions contribute to pollution with subsequent deleterious effects on the environment and health of the public [1,2]. The major routes of entry after exposure to these emissions include the respiratory system, gastro intestinal tract, the mucous membranes of the eye and the skin.

Epidemiological studies have reported impairment of lung function and increased prevalence of respiratory symptoms among workers exposed to emissions at cement plants [3,4,5]. Studies have also revealed that the respiratory system for not only the workers in cement plants, but also the surrounding community are affected [6,7]. Children in schools located within the proximity of cement plants are particularly vulnerable to cement emissions [8]. Persistent irritations of mucous membranes could lead to respiratory tract malignancies (laryngeal carcinoma) and various cancers of the intestinal tract such as colorectal, colon and stomach cancers [9,10,11]. Additionally, heavy metals have been found in urine of residents within the vicinity of cement plants [12]. Other adverse health effects due to exposure to cement emissions include skin and eye problems that lead to increased periods of hospitalization [7,13].

Given the variety of elements in the emission and its wide dispersion, effects on the mucous membranes of the eyes and nose could potentially affect the quality of life for a population in the vicinity of a cement factory. However, few studies have examined the effect of such emissions on these types of impacted communities. Additionally, most studies have investigated the effect of cement emissions on the respiratory system, especially PM that are small enough to settle in distal parts of the lungs. Zambia produces an estimated 2.2 million tons of cement annually from three cement production plants, half of which is produced at one plant, situated in Chilanga. At the edge of this plant is a settlement of 31,062, people who are potentially exposed to cement production emissions [14]. Although there has been a study into the effects of cement production on the health of workers in the factory, no such studies have been extended to the community living on the plant’s periphery. The objective of this study was to determine whether residing near a cement factory increases the risk of irritations to the mucous membrane of the eye and respiratory system.

## 2. Material and Method

### 2.1. Study Design

This was a cross sectional study was conducted in two communities; the exposed community (Freedom compound) and a control (Bauleni). The study was conducted in November and December 2013 a period characterized by wet and warm climate.

### 2.2. Study Area

**Figure 1:**
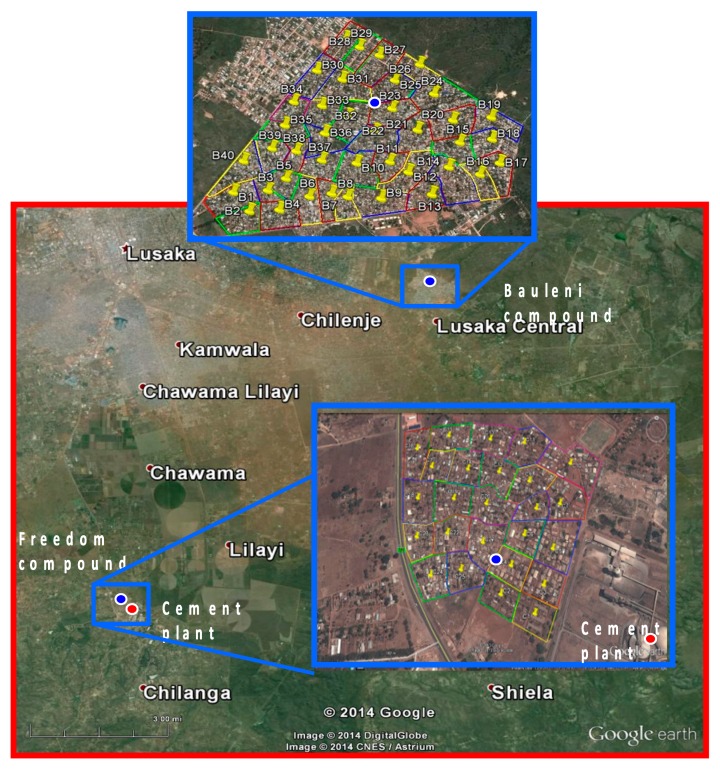
Settlements of Freedom and Bauleni.

The exposed community, Freedom, is situated in one of the most densely populated areas in Chilanga. It is located on the leeward side at the edge and to the north-west of the cement factory. It is bounded on the western side by a major intercity tarred road. Access gravel roads coming off this major road cross the breadth and width of the settlement. Traffic on the major road includes heavy trucks, buses, vans and cars. Heavy trucks rarely traverse the inner parts of the settlement. Winds across the settlement are predominantly south-westerly resulting in most traffic emissions from the main road being blown away from the settlement. The control community, Bauleni, is located about 18 km from the cement factory outside the windward cement dispersion area (Figure 1). It is bounded by major tarred roads on three sides and has minor gravel standard roads in the inside of the settlement. Traffic on the major roads and minor roads is similar to that seen in Chilanga except there are fewer heavy trucks moving on the main roads. The major economic activity is informal trade in furniture, second-hand clothes and vegetables. There are no factories within or near to the Bauleni settlement.

#### Sample Size

The prevalence of symptoms of interest in the two communities was essentially unknown. However, evidence from studies from other parts of Africa suggest that the prevalence of respiratory symptoms is around 30% for cement factory workers; while the prevalence in the control groups were found to be 10% [15,16]. To calculate our sample size for this study, the prevalence for the exposed and the control communities assumed to be similar to that found in the other studies. To detect a 20% difference at 95% confidence level and power of 80% we a required minimum sample size of 170 participants per community after adjusting for design effect of two (DE = 2) and a non-response of 30%. In this study, we targeted to recruit 220 from which we intend subsample for a future panel study.

### 2.3. Sampling of Participants

A multi-stage random sampling method was used to select participants. The study communities were each divided into geographical clusters each containing a number of households. The households in each cluster were enumerated and geocoded (see the Supplementary File). A sub-sample of 10 households per cluster was then obtained randomly. The selected households were visited and one individual from each household who met the inclusion criteria was selected and interviewed by the research assistant. Inclusion criteria were age 15–59 years and respondents must have resided in either of the study areas for at least 4 months prior to the survey. Participants employed in cement factory, construction industry, quarrying and mining were excluded.

### 2.4. Data Collection

Data were collected using a modified American Thoracic Society (ATS) questionnaire which was administered to the selected participants by trained community health workers drawn from the health facilities serving the study communities. The data collected included participants’ demographic and socioeconomic characteristics, the occurrence of symptoms of mucous membranes irritations, and exposure to tobacco smoke.

### 2.5. Measurement of Variables

Exposure variable: residence in Freedom was used as a proxy measure of exposure to cement dust and related emissions from the cement plant.

Outcomes variables: three main outcomes, eye, nose and sinus mucous membrane irritations, were assessed. Different symptoms in isolation or combination were used to define the outcomes (Table 1). Outcome variables were composite variables that were derived and a value of 1 was allocated to the variable if any of the symptoms was present and 0 if none of the symptoms was present.

**Table 1:** Symptoms used to measure the three main types of mucous membranes irritations: eyes, nose and sinus.

Symptoms or Complaints		
Eye	Nose	Sinus
Swelling	Itching	Head or face pain
Discharge	Sensation of fullness or congestion	Blowing out thick mucus
Excessive tearing	Nasal discharge	Post nasal drip **^a^**
Any of the above of symptoms **^c^**	Runny nose	Throat clearing or Hoarseness of the voice **^b^**
	Any of the above symptoms **^c^**	Any of the above symptoms **^c^**

**^a^** dripping of mucous at the back of the nose or throat. **^b^** hoarseness was defined as changes in the usual quality of the voice. **^c^** used as the main outcome (binary) in logistic regression.

Smoking status was defined as current, ex-smoker or secondary smoker. “Ex-smoker” was defined as cessation of smoking at least 1 year prior to the period study. For all categories, the cigarette was established whether it was manufactured or locally rolled tobacco (or both) how many cigarettes they had smoked per day, and for how long. “Secondary smoking” was if a participant was exposed to any household member that smoked in the house.

## 3. Statistical Analysis

Data were double entered, by two trained assistants, independently into a customised Microsoft Access database, with inbuilt validation capability. The two sets were compared, using the CompareIt program (Grig Software, Vancouver, Canada). to identify discrepancies in entries. Any discrepancies identified were checked against the paper based data. Further cleaning and coding was done in Microsoft Excel while analysis was performed using STATA version 12 (Stata Corp L 2011, College Station, TX, USA). The unit of analysis was the individual respondent. To account for multistage cluster sampling and obtain correct estimates, STATA was set to *svy mode*, setting the primary sampling unit as the cluster of households.

Univariate analysis was done to describe the distribution of respondents’ demographic and socioeconomic characteristics within and between the exposed and control communities: proportions, median and inter-quartile range (IQR) and 95% confidence intervals were reported. For categorical data the Pearson’s Chi-square test was used to compare differences between the communities. All statistical tests were two-sided and a *p*-value < 0.05 was considered as statistically significant while *p*-value ≥ 0.05 and ≤ 0.1 were considered marginally statistically significant.

To examine the strength of association between area of residence and each of the three outcomes, bivariate and multivariable logistic regression was used to obtain unadjusted and adjusted odds ratios (ORs), *p*-values, and their respective 95% CI. The following factors were assessed: area of residence, age, gender, marital status, education, occupation, whether respondent ever smoked (and number of pack years smoked), source of energy for cooking and lighting, whether cooking area was located within the main house or sleeping area; and ventilation of the dwelling house and whether respondent spent time home or away from home. For categorical factors, dummy variables were used in the model selection procedure. Furthermore, statistical interactions between community and other factors were investigated.

Three multivariable models, one for each outcome, were utilized. To obtain adjusted ORs for effect of “area of residence” on the outcomes all potential confounders, (*i.e.*, factors with a *p*-value < 0.05 in bivariate analysis) were placed in an initial logistic regression model. This was followed by the addition, in stepwise manner, of variables that were marginally significant in bivariate analyses. Each time a new factor was added to the model, the ORs of the factors already in the model were checked. If the addition of a new factor changed the OR of any already included variable by more than 10%, the additional variable was retained in the final multivariate model otherwise the variable was removed and a different one added. Area of residence was considered the main explanatory variable and therefore was included in all models for each outcome of interest regardless of whether it was statistically significant in bivariate analyses.

## 4. Results

### 4.1. Description of Respondents’ Demographic and Socioeconomic Characteristics

In total, 423 respondents took part in the study; 225 and 198 from exposed and control communities, giving a response rate of 100% and 90% respectively. Table 2 and Table 3 summarize the demographic and socioeconomic characteristics of the respondents stratified by community.

The age distribution and gender was significantly different between the two communities. While 46.2% of respondents in Freedom were in the 25 to 39 years age group, 39.5% were younger (12–25 years of age) in Bauleni. Although the majority of respondents in both communities were female, there was a significant difference between the community; 84.1% and 73.2% in Freedom and Bauleni respectively. The median number of years respondents lived in each community as well as the marital status distribution was not different. There were more (*p* = 0.003) unemployed respondents in Freedom (75.5%) than Bauleni (61.6%).

The majority of respondents from both communities had never smoked, with only 23 respondents in the two communities reporting having ever smoked (Table 2). Of these, six were ex-smokers while 17 were current smokers. Overall, there was no statistical difference in smoking status; between the two communities. Respondents pack years smoked ranged from 5 to 35 years for the whole group of smokers.

### 4.2. Socio Economic Characteristics of the Communities

Although, the majority of houses in both communities were made of concrete material, Bauleni compared to Freedom, had a significantly higher proportion (*p*-value = 0.020) (Table 3). The majority of houses in Freedom were roofed with asbestos while metal sheets were used for most houses in Bauleni. A statistically significant higher proportion of houses in Freedom were plastered (*p* value = 0.010). No statistically significant differences in the distributions of number of rooms and windows were observed between the communities. Most houses in both communities had up to two rooms and up to three windows per structure with no statistically significant difference. Only a minority of houses owned a floor carpet in the two communities; with no statistical significant difference between communities (*p* value = 0.260).

While the source of energy for lighting was the same for both communities, energy source for cooking was statistically significantly different between the communities; majority of households in Freedom used charcoal as source of energy for cooking and had cooking areas located within the dwelling house.

**Table 2:** Description of study participants by demographic characteristics stratified by community.

Factor	Total	Freedom	Bauleni	*p-Value*
		(Exposed)	(Control)	
	*N* = 423	*N* = 225	*N* = 198	
	n (%)	n (%)	*n* (%)	
Age in years:				
12–24	158 (37.4)	77 (33.5)	81 (39.5)	0.005
25–39	166 (39.2)	101 (46.2)	65 (31.7)	
40+	99 (23.4)	47 (20.4)	52 (28.8)	
Years lived in community **^a^**		*N* = 217	*N* = 190	
*median (IQR)*		10 (4–21)	14 (5–23)	0.080
Gender				
*Female*	333 (78.7)	187 (83.1)	146 (73.7)	0.021
*Male*	90 (21.3)	38 (16.9)	52 (26.3)	
Marital status				
*Single*	138 (32.6)	71 (36.6)	67 (32.8)	0.099
*Married*	245 (57.9)	135 (57.9)	110 (57.2)	
*Widow/divorced*	40 (9.5)	19 (5.5)	21 (10.0)	
Education				
*None*	28 (6.6)	4 (1.1)	24 (9.6)	
*primary*	241 (57.0)	147 (63.9)	94 (49.7)	<0.001
*Secondary*	145 (34.3)	66 (30.8)	79 (40.3)	
*Tertiary*	9 (2.1)	8 (4.2)	1 (0.4)	
Employment status **^b^**				
*Unemployment*	270 (67.0)	153 (75.5)	117 (61.6)	0.003
*Employed*	133 (33.0)	56 (24.5)	77 (38.4)	
Smoking status **^c^**				
*Never smoked*	397 (94.7)	209 (94.5%)	188 (96.6%)	0.381
*Ever smoker*	6 (1.4)	5 (2.0%)	1 (1.0%)	
*Current*	17 (4.0)	10 (3.5%)	7(2.8)	
*Secondary smoke*				
*Yes*	24 (5.7)	9 (3.2)	15 (7.6)	0.060
*No*	395 (94.3)	215 (96.8)	180 (92.4)	

**^a^** missing values 8 and 8 for Freedom and Bauleni respectively. **^b^** missing values 16 and 4 for Freedom and Bauleni respectively. **^c^** missing values 1 and 3 for Freedom and Bauleni respectively.

**Table 3:** Description of respondents by social economic status stratified by community.

Factor	Total	Freedom	Bauleni	*p-Value*
		(Exposed)	(Control)	
	*N* = 423	*N* = 225	*N* = 198	
	*n* (%)	*n* (%)	*n* (%)	
House ownership				
*Owned*	180 (42.6)	85 (44.2)	95 (53.9)	
*Rented*	224 (52.9)	124 (49.4	100 (44.8)	0.021
*Other*	19 (4.5)	16 (6.2%)	3 (1.3)	
How old house Yrs **^a^**				
*1*–*20*	70 (16.9)	33 (16.8)	37 (20.4)	
*21*–*40*	23(5.5)	6 (3.9)	17 (9.4)	0.080
*Unknown*	322 (77.6)	184 (79.3)	138 (70.1)	
House material **^b^**				
*Mud*	49 (12.3)	36 (16.5 )	13 (6.6)	0.020
*Concrete*	351 (87.5)	171 (76.5)	180 (90.9)	
Roof material **^c^**				
*Metal*	191 (45.9)	55 (24.1)	136 (71.5)	<0.001
*Asbestos*	225 (54.1)	167 (75.9)	58 (28.5)	
House plastered				
*Yes*	205 (48.5)	130 (58.8)	75 (38.5)	0.010
*No*	213 (50.4)	90 (48.0)	123 (123)	
No. of rooms **^d^**				
*1*–*2*	240 (58.4)	123 (50.8)	117 (53.5)	0.530
*3+*	117 (41.6)	92 (49.2)	79 (46.5)	
No. of windows **^e^**				
*0*–*3*	305 (73.0)	158 (64.6)	147 (68.9	0.420
*>=4*	113 (27.0)	64 (35.4)	49 (31.1)	
Carpet in house				
*Yes*	121 (28.6)	58 (25.0)	63 (31.3)	0.260
*No*	302 (71.4)	167 (75.0)	135 (68.7)	
Kitchen location				
*Outside*	225 (53.2)	102 (46.5)	123 (64.6)	0.004
*insideInside*	198 (46.8)	123 (53.5)	75 (35.4)	
Source energy cook				
*Electricity*	199 (47.0)	82 (35.3)	117 (59.0)	<0.001
*Charcoal*	224 (53.0)	143 (64.7)	81 (41.0)	
Source energy light **^f^**				
*Electricity*	288 (73.5)	149 (72.3)	139 (73.4)	0.830
*Candle*	104 (26.5)	51 (27.7)	53 (26.6)	

**^a^** 2 and 6 values missing for Freedom and Bauleni respectively. **^b^** 18 and 5 missing values for Freedom and Bauleni respectively. **^c^** 3 and 4 missing values for Freedom and Bauleni respectively. **^d^** 10 and 2 values missing for Freedom and Bauleni respectively. **^e^** 3 and 2 missing values for Freedom and Bauleni respectively. **^f^** 25 and 6 missing values for Freedom and Bauleni respectively.

### 4.3. Irritations of Mucous Membranes of Eyes, Nose and Sinus

Generally, the prevalence of constituent symptoms of either eye, nose or sinus membrane irritations were significantly higher in Freedom compared to the control community (Table 4). Larger proportions of respondents in Freedom reported “any combination of symptoms” compared to Bauleni; 78.2% *versus* 49.9%, 66.9% *versus* 29.4% and 73.7% *versus* 53.3% for eye, nasal and sinus irritations, respectively (*p* value < 0.001). Itching and tearing, runny nose and fullness; and headaches and clearing were the most common reported symptoms for eye, nasal and sinus irritations, respectively, in the two communities.

**Table 4:** Proportions of respondents reporting the individual and constituent symptoms for irritations of eye, nose and sinus mucous membranes irritation by community.

	*N* = 420	*N* = 223	*N* = 197	*p-Value*
	*n* (%)	*n* (%)	*n* (%)	
Eye irritation				
*Itching*				
*Yes*	163(38.8)	126 (56.4)	37 (18.3)	<0.001
*Swelling*				
*Yes*	82 (19.5)	53 (26.1)	29 (13.7)	0.007
*Discharge*				
*Yes*	66 (15.7)	49 (20.7)	17 (10.3)	0.020
*Tearing*				
*Yes*	122 (29.0)	87 (42.8)	35 (18.9)	<0.001
*Any of the symptoms*				
*Yes*	268 (63.8)	170 (78.2)	98 (49.9)	<0.001
Nose irritation				
*Itching*				
*Yes*	83 (19.8)	53 (25.1)	30 (14.6)	<0.001
*Fullness*				
*Yes*	95 (22.6)	56 (25.0)	39 (17.0)	0.012
*Nasal discharge*				
*Yes*	62 (14.7)	38 (17.0)	24 (9.8 )	0.030
*Runny nose*				
*Yes*	115 (27.4)	74 (35.2)	41 (19.8)	<0.001
*Any of the symptoms*				
*Yes*	206 (49.0)	141 (66.9)	65 (29.4)	<0.001
Sinus irritation				
*Head pain*				
*Yes*	188 (44.8)	119 (54.6)	69 (38.4)	<0.003
*Thick mucus*				
*Yes*	67 (16.0)	40 (21.4)	27 (11.6)	0.002
*Post nasal drip*				
*Yes*	72 (17.1)	44 (21.3)	28 (12.8)	0.040
*Throat clearing*				
*Yes*	92 (21.9)	55 (26.9)	37 (16.5)	<0.004
*Any of the symptoms*				
*Yes*	260 (61.9)	157 (73.7)	103 (53.3)	<0.001

### 4.4. Predictors of Mucous Membrane Irritations

Area of residence, time where respondents spent most of the time, location of the cooking area or kitchen and source of energy for cooking were significant predictors of eye irritation in Bivariate analyses (Table 5). After adjusting for time where respondents spent most of the time, location of the kitchen and source of energy for cooking, respondents in Freedom were 2.50 (95% CI (1.65, 3.79)) times more likely to have eye irritation than those in the control community. Respondents who spent time around their home were 1.8 times more likely to have eye irritations controlling for other factors. However, when stratified by area of residence there was evidence of effect modification; respondents from Freedom who spent time around home were 2.8 times more likely to experience eye irritations while respondents of Bauleni were only 1.7 times more likely to experience eye irritations.

In Bivariate analyses, residence, age, gender, and source of energy for cooking were found as significant predictors for nose irritation. Although result not included in Table 5, duration of living in the community showed effect modification; living in Freedom community increased the odds of nose irritation by 1% (OR 1.01, *p*-value = 0.008) while it had no effect for respondent living in Bauleni (OR 1.00, *p*-value = 0.683). In multivariate models only residence, age and gender retained statistical significance. Compared to respondents from the control community, respondents from the exposed community were 4.36 (95% CI (2.96, 6.55)) times more likely to have nose irritation.

Independent determinants of sinus irritations included area of residence, age, education, and occupational status, source of energy for cooking and presence of floor carpet (Table 5). However, only residence, age and occupation retained statistical significance after adjusting for the other predictors. Respondents from the exposed community were 1.94 (95% CI (1.19, 3.18)) more likely to have sinus irritation compared to those from the control community adjusting for confounders. Duration of living in the exposed community showed no effect on risk of sinus irritations.

## 5. Discussion

The study investigated the prevalence and determinants of mucous membrane irritations among residents of a community residing near a cement factory and a control community in the area of Lusaka, Zambia. Prevalence of all mucous membrane irritations were higher in the exposed compared to the control community and residence in the exposed community was a strongly significant determinant of all types of mucous membrane irritations.

The excessive prevalence of all types of mucous membrane irritations in the exposed community compared to the control could be attributed to increased exposure to chemical and particulate matter irritants in the ambient air. For instance excessive tearing and itching of the eye; nasal itching and fullness; and head pain and throat clearing are all common manifestations when mucous membranes of the eyes, nose and/or the sinuses are exposed to chemical irritants. Literature shows that irritations of the eyes due to exposure to chemicals and particulate matter often manifest as excessive tearing with or without itching, while swelling or discharge, which were less often reported, is often related to infections of the eyes [17,18,19].

**Table 5:** Significant factors associated with outcomes in Bivariate and Multi variate- analyses.

Site of Irritation	Independent Factors	Crude ORs	*(95% CI)*	*p*-Value	Adjusted ORs	*(95% CI)*	*p*-Value
Eye irritation	Community						
	*Bauleni*	1	-	-	1	-	-
	*Freedom*	3.60	2.56–5.28	<0.001	2.50 ^a^	1.65–3.79	<0.001
	Time spent						
	*Away home*	1	-	-	-	-	
	*Around home*	2.45	1.63–3.70	<0.001	1.78	1.12–2.82	0.017
	Kitchen Location						
	*Outside*	1	-	-	-	-	
	*Inside*	1.56	1.12–2.18	0.012	1.62	1.12–2.34	0.013
	Cook energy						
	*Electricity*	1	-	-	-		
	*Charcoal*	1.70	1.06–2.71	0.028	1.55	1.06–2.28	0.003
Nose irritation	Community						
	*Bauleni*	1	-		1	-	-
	*Freedom*	4.83	3.15–7.41	<0.001	4.36 ^b^	2.95–6.55	<0.001
	Age (years)						
	*12*–*24*	1	-	-	-	-	-
	*26*–*39*	0.99	0.64–1.51	0.964	0.73	0.46–1.13	0.155
	*40+*	0.58	0.33–1.01	0.057	0.60	0.36–0.01	0.053
	Gender						
	*Female*	1	-	-	1	-	-
	*Male*	0.41	0.23–0.78	0.006	0.47	0.25–0.85	0.017
	Cook energy						
	*Electricity*	1			-		
	*Charcoal*	1.80	1.11–2.96	0.021	1.37	0.82–2.31	0.214
Sinus irritation	Community						
	*Bauleni*	1	-	-	1	-	-
	*Freedom*	2.45	1.57–3.83	<0.001	1.94 ^c^	1.19–3.18	0.012
	Age (years)						
	*12*–*24*	1	-	-	-	-	-
	*25*–*39*	0.53	0.28–1.00	0.052	0.46	0.21–0.03	0.044
	*40+*	0.70	0.34–1.43	0.311	0.83	0.36–1.9	0.595
	Education						
	*None*	1	-	-			
	*Primary*	2.41	0.98–5.97	0.055	1.98	0.66–5.88	0.202
	*Secondary*	1.46	0.63–3.37	0.350	1.25	0.36–4.26	0.703
	*Tertiary*	2.66	0.61–11.53	0.178	2.54	0.73–8.82	0.132
	Occupation						
	*Unemployed*	1					
	*Unemployed*	0.56	0.35–0. 89	0.019	0.69	0.47–0.99	0.048
	Cook energy						
	*Electricity*	12.13	-	-	-		
	*Charcoal*	2.13	1.22–3.71	0.011	1.68	0.88–3.22	0.108
	Floor carpet						
	*No*	1	-		-	-	
	*Yes*	0.55	0.35–0.87	0.013	0.66	0.38–1.14	0.129

**^a^** adjusted for time spent around home, kitchen location and source of energy for cooking. **^b^** adjusted for age, gender and source of energy for cooking. **^c^** adjusted for age, education, occupation, source of energy for cooking and presence of floor carpet in the dwelling house.

Merhaj *et al.* demonstrated similar findings when they reported that 97% of the respondents in a community within the vicinity of a cement factory suffered from eye irritations [20]. Additionally, nasal itching and fullness of the nostrils are often associated with allergic responses to specific, non-infectious particles such as plant pollens, dust mites, animal air, industrial chemicals and medicines, while nasal discharge is often due to infectious or foreign lodgment in the nasal cavity [18,19]. Furthermore, head pain and throat clearing have been demonstrated to be common manifestations of sinus irritation [21,22]. Residence in the exposed community was a common and most significant determinant of all the types of mucous membranes irritations in this study; increased the odds of suffering from irritation by 2.4, 3.6 and 4.8 times for sinus, eye and nasal irritations respectively. The increased risk is associated with exposure to various emissions during cement production among the residents of Freedom compound. Although direct measurements of emissions in Freedom community were not performed, residence in this community was considered an adequate proxy measure of exposure as other studies have demonstrated high levels of PM, NOx and CO in ambient air in communities near cement factories [1,2].

The likelihood of eye irritations was affected by various other factors. Respondents that spent more time around the home had higher odds of eye irritations after controlling for area of residence and other confounders. Studies have shown that human health risks relate to specific pollutants, their concentration and to exposure as a function of time spent in the contaminated environment [23]. In this study, stratifying the analysis by area of residence showed slight effect modification as respondents from the exposed, compared to the control community, were more likely to experience eye irritations. This finding, suggests higher risks of eye irritation due to higher contamination levels in the environments of the exposed community. Several studies have shown excess risk for eye irritation among exposed individuals, either in community or factory settings [4,13]. However, it is also possible that others factors of the domestic micro-environment that were not considered in this study contributed to these findings.

Using charcoal as a source of energy for cooking was associated with 55% increase in eye irritations for the exposed group. The health effects associated with exposure to emissions of unclean energy fuels have been established. Studies have consistently demonstrated eye irritations ranging from reddening, itching, watering and discomfort to be the most common response to exposure to emissions from combustion of unclean energy such as charcoal [23,24]. Contrary to literature, using “dirty fuels” as source of energy for lighting did not attain statistical significance in the current study. This could in part, be due to the large number of respondents in both communities using electricity for lighting. Related to energy source, respondents whose kitchen location was located inside the main house were more likely to experience eye irritation. Cooking within the same area used for sleeping exposes the eyes to excessive emissions from the cooking process emissions such as the ultrafine particles [25]. Medical literature shows that the eye is a sensitive organ that easily gets irritated when subjected to any of irritants [17].

Gender was the only factor that was associated with nasal irritation in multivariable model other than community. Females, as revealed in other studies, tend to be more prone to nasal irritation [26,27]. These differences could be due to cooking on open fires, exposure to chemicals found in household cleaning agents and other factors that were not measured in the study.

Community, age, education, occupation, source of energy for cooking and presence of carpet were independently associated with sinus irritation in bivariate analyses, but only residence, age and occupation retained statistical significance after adjusting for other predictors. Contrary to what has been found in studies in industrialized communities [28], floor carpet was not a risk factor in this study. This could partly be explained by respondents’ behavior; residents in the studied communities spend most of their time outside their homes such that the effect of floor carpet could not be seen. Additionally, the proportion of respondents owning a carpet was small and could not have given statistical power to detect a small difference.

Weaknesses of this study should be highlighted in interpreting the results. Although the study provides evidence of differences in the prevalence of mucous membrane irritations in the communities, it cannot explain the cause of the difference. This is mostly due to the inherent weakness of cross sectional epidemiological study designs in providing evidence of causation or associations. The study did not measure PM, NOx, SOx and any other possible pollutant in the ambient air; neither did it ascertain the source apportionment of these substances. It is therefore difficult to state, with certainty, that the observed differences in prevalence of irritations in the two communities were due to the presence of emissions from the cement plant. It is possible that there were other sources of pollution in the exposed community that the study did not account for. Furthermore, information regarding allergic tendencies, which are possible causes of symptoms of mucous membrane irritation, was not collected thus limiting interpretations to a certain extent.

Additionally, symptoms were self-reported and not verified with hospital records. Self-reporting could have introduced recall bias especially that has been much media publicity about the adverse effects the cement plant has had on the environment and people in the vicinity of the plant [29]. Therefore, respondents from the exposed community could have exaggerated the occurrence of respiratory problems. There was a likelihood of misclassification of employment status and exposure as most respondents could not accurately describe their occupational tasks. Finally, the results may not be generalisable to both sexes in the study since the proportion of female respondents was far more than would be expected in the general population [30].

## 6. Ethical Considerations

The study protocol was reviewed and approved by a local research ethics committee in Zambia-ERES Converge IRB (00005948) and from IRBs of the Universities of Pretoria (0000 2535 IORG 0001662) and Michigan (00070842).

## 7. Conclusions

Irritations to mucous membrane of the eyes, nose and sinuses are common but prevalence is increased several fold in the presence of air pollution. This study shows that residence within the vicinity of a cement production plant increase the odds of experiencing these irritations. Cohort panel studies to investigate mucous membrane irritations and respiratory symptoms coupled with field characterization of exposure to air pollutants are needed to assess causality.

## Supplementary Files

Supplementary File 1
