# Supplementary material for: Prevalence and Determinants of Mucous Membrane Irritations in a Community Near a Cement Factory in Zambia: A Cross Sectional Study
Source: Int J Environ Res Public Health. 2015 Jan 16;12(1):871–87. doi: 10.3390/ijerph120100871 (PMC4306898; doi:10.3390/ijerph120100871)
Supplement: Supplementary File 1 [file ijerph-12-00871-s001.pdf]

Supplementary Information

Prevalence and Determinants of Mucous Membrane Irritations in a Community Near a Cement Factory in Zambia: A Cross Sectional Study

A. Selection of Study Participants (Geocoding)

Study participants were selected using multi-stage random sampling approach. The first tier of sampling frame was obtained by dividing each of the two communities into clusters of households using the latest Google Earth maps of the two communities (Figures S1 and S2).

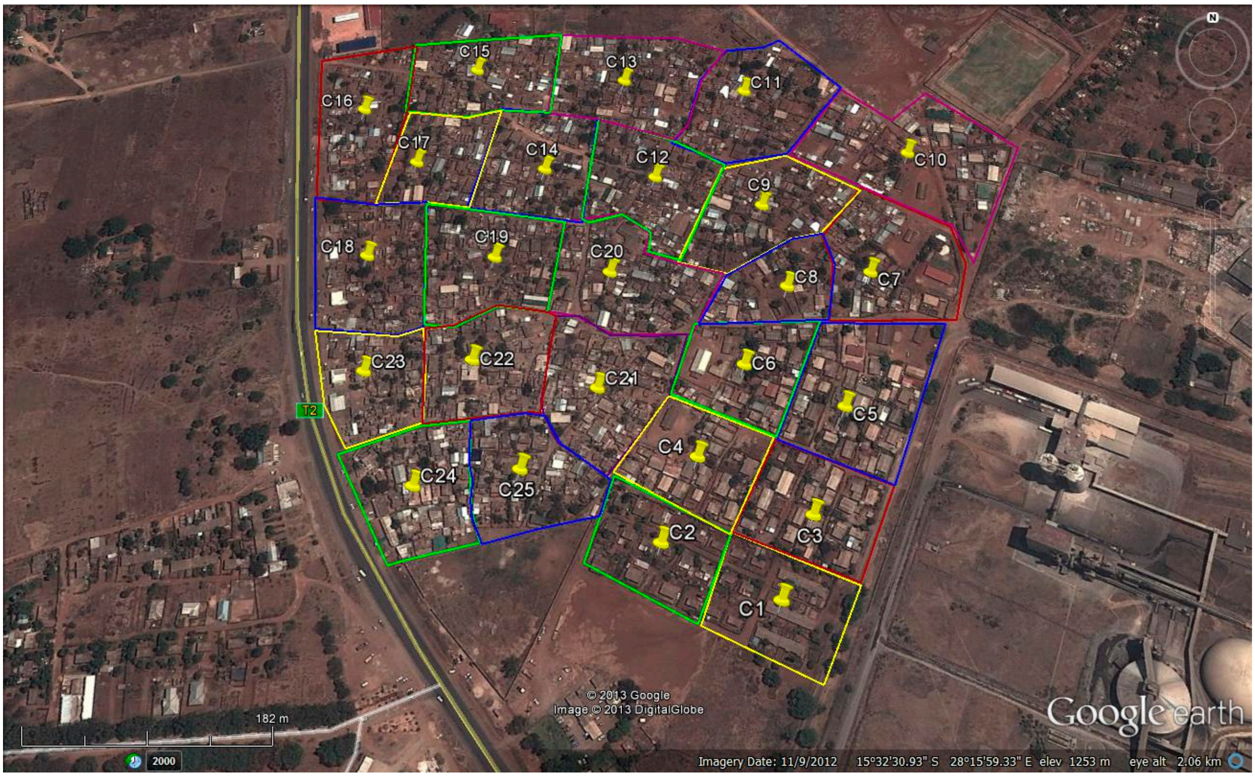

Figure S1. Freedom compound.

The cluster size was determined depending on geophysical arrangement of the households, e.g., division by main roads, markets, schools or other geographical features like a stream or river. As there was no pre-enumeration of households in both communities, attempts at getting equal number of households per cluster was ensured by making clusters of roughly equal geographical dimensions. This resulted in 25 and 42 clusters in Freedom and Bauleni respectively.

This was followed by identification and physical mapping of the clusters using GPS coding. The delineated clusters for each community were then numbered sequentially and entered into Microsoft Excel to form the first tier of sampling frame (one for each community). A subset of 10 clusters from each sampling frame was randomly chosen using random number generator in Excel. Excel was set to generate random number between 0 and 1using the “RAND ()” function. Two empty columns were created adjacent to the column listing the clusters. Random numbers were generated in

one column. These were copied and pasted, as values, in the other empty column. The values were then sorted and the first ten numbers corresponding to 10 clusters were selected [1]. This ensured that the clusters were selected at random and representative of the community.

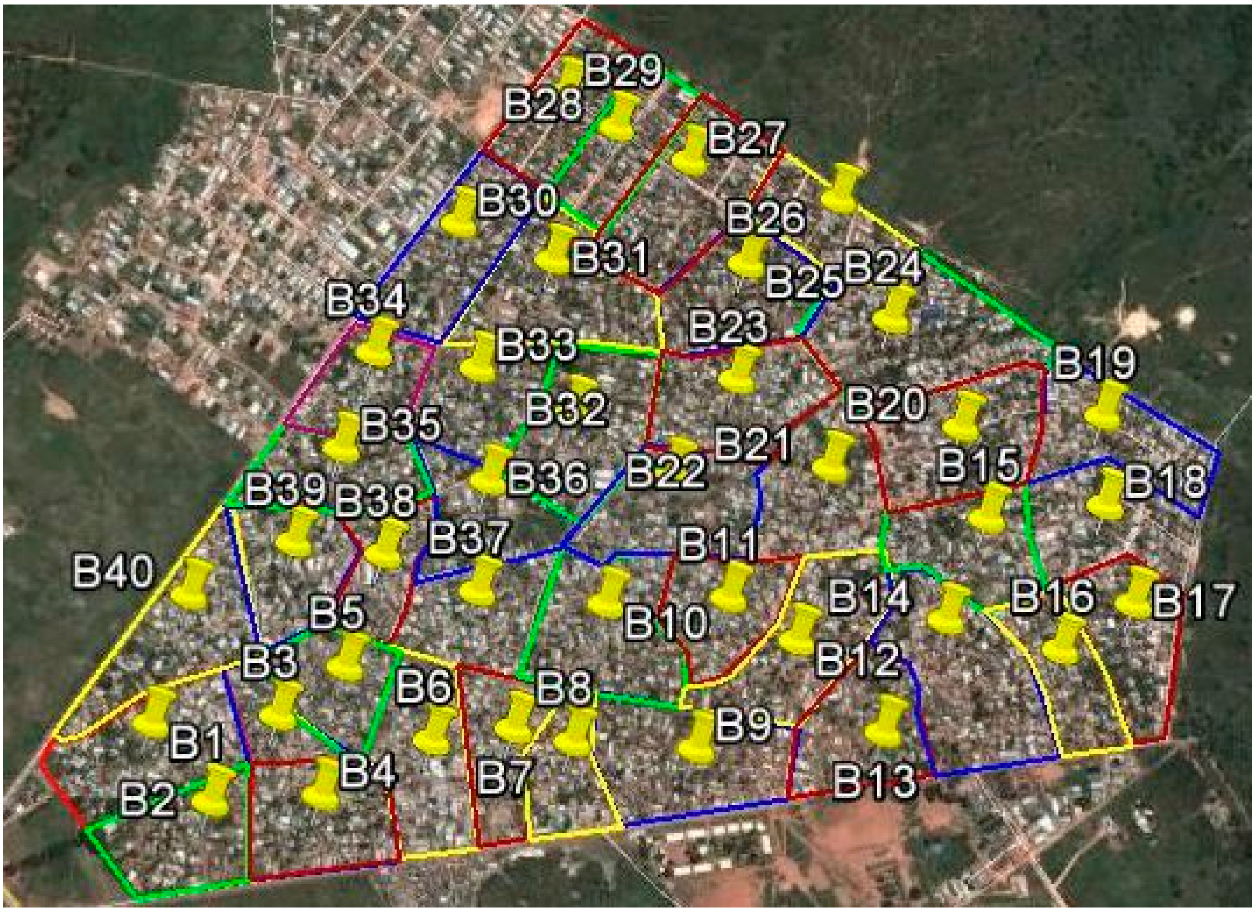

Figure S2. Bauleni compound.

The second tier of sampling frame comprised all households in the selected clusters. Each household in each selected cluster was numbered using GPS geocodes (and house numbers where possible). Separate sampling frames for each cluster was entered in Microsoft Excel and a random subsample of 22 households was obtained following the method described in the previous section. A total of 220 households were thus selected for each community. This was done to ensure the included households were chosen at random and were representative of the community. The chosen households were then visited, aided by the geocodes and house numbers, to determine household eligibility.

Consent was sought from the head of each household to participate in the study. Individuals who met the inclusion criteria and gave consent were numbered sequentially on small pieces of paper, which were put in a bag and one selected by lottery. This was followed by administration of a questionnaire. Where there was only one individual meeting the inclusion criteria in the household, that individual was chosen for inclusion in the study.

In the event that the head of household declined to participate, the entire house then became ineligible, the next available household was chosen randomly. This was done by numbering clockwise the immediate surrounding households on pieces of paper. Then the pieces of paper bearing the

numbers were put in a box, shaken and one paper drawn at random. The chosen household replaced the one that did not meet the inclusion criteria in the initial sampling. If the replacement household did not meet the inclusion criteria, the procedure was repeated. In the repeat sampling all households that did not meet the inclusion criteria were excluded.

B. Questionnaire: Does Exposure to Cement Emission Cause an Increased Risk of Mucous Membranes Irritation: a Cross Sectional Study

Demography Information

1. Gender

   Male ☐
   Female ☐

2. What is the ethnic group of the household?

   1. Black ☐
   2. Other ☐ Specify……………………………..

3. What is your marital status

   1. Married ☐
   2. Single ☐
   3. Divorced ☐
   4. Widowed ☐

4. What is your age at your last birthday? ☐☐
5. What is your highest Level of education you have attained?

   1. Primary ☐
   2. secondary ☐
   3. Tertiary ☐
   4. None ☐

6. For how long have you lived in this community?

   …………Months…………Years

7. How many people usually live in this household? ☐
8. How many residences have you changed in the last 10 years?

   …………………………

9. Are you likely to shift from this community in the next four moths

   Yes ☐ No ☐

Social Economic Status

10. How many rooms does your house have? (Do not take into account the cooking areas if it is not within the main structure)

    ………………………rooms

11. The house where you live, is it?

    a. Owned ☐
    b. Rented ☐
    c. Borrowed ☐
    d. Other (specify)……………………………………………..

12. How old (approximate years) is your house?

    a. ………years
    b. Not known

13. How many persons provide economic support for the house

    …………… Person (s)

14. What is your occupation?

    1. Bricklayer ☐
    2. Housewife ☐
    3. Farm worker ☐
    4. Student ☐
    5. Other ☐ (Specify)……………………………………………

15. Do you own any of the following appliances or vehicles

a. Radio	yes	☐	No	☐
b. Television	Yes	☐	No	☐
c. Refrigerator	Yes	☐	No	☐
d. Bicycle	Yes	☐	No	☐
e. Motorcycle	Yes	☐	No	☐
f. Car	Yes	☐	No	☐

(Q16 to 22, ask the following and observe)- Household structural characteristics

16. How many rooms does the house have:

    1. ☐
    2. ☐
    3. ☐
    4. ☐
    5. Others ☐ (Specify)……………………………

17. Number of windows in the house?

    1. ☐
    2. ☐
    3. ☐
    4. ☐
    5. Others ☐ (Specify)……………………………………………………

18. Number of the doors in the house?

    1. ☐
    2. ☐
    3. ☐
    4. ☐
    5. Others ☐ (Specify)……………………………

19. What material is the house made of? (Observe)

    1. Pan bricks ☐
    2. Mud bricks ☐
    3. Concrete blocks ☐
    4. Metal sheets ☐
    5. Other ☐ Specify……………………………

20. What is the wall finish of the house?

    1. Plastered ☐
    2. Un-plastered ☐

21. What is the roof made of?

    1. Grass thatched ☐
    2. Metal sheets ☐
    3. Asbestos sheets ☐
    4. Plastic ☐
    5. Other ☐ Specify……………………………………………………

22. Is there a floor carpet in the house?

    1. Yes ☐
    2. No ☐

Determinants of exposure

23. How is the kitchen located in reference to main house?

    1. Within main house ☐
    2. Separate from main house in an enclosed structure ☐
    3. Outside in an open space ☐ ***(Skip to Q25)***

24. If cooking is done within main house, is it done in the room where you sleep?

    1. Yes ☐
    2. No ☐

25. What is the main source of energy for cooking?

    1. Electricity ☐
    2. Charcoal ☐
    3. Firewood ☐
    4. Rechargeable lamp ☐
    5. Other ☐ (Specify)……………………………….

26. What is the source of energy for lighting?

    1. Electricity ☐
    2. Firewood ☐
    3. Paraffin lamp ☐
    4. Others ☐ (specify) ………………………………

27. Form of ventilation? Tick all that applies for each room

Room	Through Ventilation	Cross Ventilation	None
1			
2			
3			
4			
5			
6			
7			
8			
9			
10			

28. Measure size of the window in relation to floor area? {**Excluding toilet and bathroom**}

Room	Floor Area	Size of Window
1		
2		
3		
4		

Questions on Lifestyle

29. Do you smoke or have you ever smoked cigarette?

    1. Never smoked ☐ **Skip to 36)**
    2. Ex-smoker ☐ **(Complete Qs 31-32)**
    3. Smoke now ☐ **(Skip to Q33)**

30. If **ex- smoker**, how long ago did you quit smoking?

    ………… Months…… Years

31. If ex-smokers, how many years did you smoke before you quit smoking?

    ………… years

32. How many cigarettes were you smoking per day?

    ……………… Cigarettes

33. If **current**, what type of cigarette do you smoke?

    1. Factory manufactured cigarette? ☐
    2. Locally rolled tobacco? ☐
    3. Both ☐

34. If **current smoker**, approximately how many cigarettes a day do you smoke?

    ☐

35. How long have you been smoking?

    ☐☐

36. Does any of the household members smoke?

    Yes ☐ No ☐

37. Do they smoke in the house?

    Never ☐
    Occasionally ☐
    Almost every day but only a few cigarettes (up to 2) ☐
    Almost daily many cigarettes (more than 2) ☐

38. Where do you spend most of your time?

    Away from home ☐
    Around home/inside home ☐
    Other ☐ (specify) ……………………………

39. How do you spend most of your time during the day?

    1. In and around the home ☐
    2. Away from home, but within Chilanga/Bauleni ☐

3. Away from home, out of Chilanga/Bauleni ☐
(specify) ……………………………

Respiratory health symptoms

40. Do you experience any of the following with your eyes? (tick all that applies**[PROMPT])**

a. Itching ☐
b. Swelling ☐
c. Discharge ☐
d. Excessive tearing ☐

41. Do you experience any of the following with your nose? (tick all that applies)

a. Itching ☐
b. Sensation of fullness, congestion ☐
c. Blockage ☐
d. Discharge or runny nose ☐

42. Do you experience any of the following with your sinuses (tick all that applies)

a. Head or pain in the face ☐
b. Blowing out thick mucus ☐
c. Postnatal drip in the back of throat ☐
d. Throat clearing or hoarseness of voice ☐

Cough

43. Do you usually have a cough? (Count a cough with smoke or on first going out-of-doors. Exclude clearing of throat)

Yes ☐ No ☐ ***(If NO skip to question 45)***

44. Do you usually cough as much as 4 times to 6 times a day, 4 or more days out of the week?

Yes ☐ No ☐

45. Do you usually cough at all on getting up, or first thing in the morning?

Yes ☐ No ☐

46. Do you usually cough at all OR during the rest of the day OR at night?

Yes ☐ No ☐ ***If Yes to any 43,44, 45 or 46, ASK 47 and 48***

47. Do you usually cough like this on most days for 3 consecutive months or more during the year?

Yes ☐ No ☐

48. For how many years have you had this cough?

……… days
…………….. Does not apply

Phlegm

49. Do you usually bring up phlegm from your chest? (Count phlegm with the first smoke or going out-of-door. Excluding from nose. Count swallowed phlegm) ***[if NO skip to 54]***

    Yes ☐ No ☐ ***(In NO skip to 51)***

50. Do you usually bring up phlegm as much as twice a day, 4 or more days out of a week?

    Yes ☐ No ☐

51. Do you usually bring up phlegm at all on getting up, or first thing in the morning?

    Yes ☐ No ☐

52. Do you usually bring up phlegm at all during the rest of the day or at night?

    Yes ☐ No ☐

If YES to any of the above (49, 50, 51 or 52) ASK 52 and 53: If NO to all, skip to 55

53. Do you bring phlegm like this on most days for 3 consecutive months or more during the year?

    Yes ☐ No ☐

54. For how many years have you had this cough?

    ……………………. years
    …………………….. Does not apply

Episodes Of Cough

55. Have you had periods or episodes of increased cough or phlegm lasting 3 weeks or more year (**for persons who usually have cough)**

    Yes ☐ No ☐ ***(If NO Skip, GO to Q56)***

56. For how long have you had at least 1 (one) such episode?

    ……………….. Number of years
    ………………. Does not apply

Episodes of Phlegm

57. Have you had periods or episodes of increased phlegm lasting 3 weeks or more each year **(for persons who usually have phlegm)**

    Yes ☐ No ☐ Does not apply ☐ (***Skip to Q58)***

58. For how long have you had at least 1 (one) such episode?

    ……………….. Number of years
    ………………. Does not apply

Wheezing

59. Does your chest ever sound wheezing or whistling **(Tick all that applies)**

    1. when you have a cold Yes ☐ No ☐
    2. occasionally apart from colds Yes ☐ No ☐
    3. most days or nights apart from a cold Yes ☐ No ☐

If YES, to 1, 2, or 3 in Q59; ASK Q 60. If NO skip, GO to Q61, Q62 and Q63

60. For how many years has this been present

    …………… year (s)
    …………… does not apply

61. Have you ever had an attack of wheezing that has made you feel out of breath?

    Yes ☐ No ☐ ()

62. How old were you when you had your first attack?

    ………… years

63. Have you ever required medicine or treatment for the(se) attack(s)

    Yes ☐ No ☐

Breathnessness

64. Are you troubled by shortness of breath when hurrying on the level or walking up a slight hill?

    Yes ☐ No ☐ ***(If NO skip, Go to Q69)***

If Yes in Q64 ASK Q65, Q66, Q67 and Q68

65. Do you have to walk slower than people of your age on the level because of breathless?

    Yes ☐ No ☐ Does not apply ☐

66. Do you ever have to stop for a breath when walking at your pace on the level?

    Yes ☐ No ☐ Does not apply ☐

67. Do you ever have to stop to breathe after walking a few minutes on the level?

    Yes ☐ No ☐ Does not apply ☐

68. Are you breathless to leave the house or breathless on dressing or undressing?

    Yes ☐ No ☐ Does not apply ☐

Chest Cold And Chest Illness

69. If you get a cold, does it usually go to your chest (usually more than ½ the times?)

    Yes ☐ No ☐ Don't get colds ☐

70. During the past 3 years, have you had any chest illness that kept you from work or school, or in doors at home, or in bed?

    Yes ☐ No ☐

If YES to Q70 ASK Q71 and 72, IF NO skip to 73

71. Did you produce phlegm with any of these chest illnesses

    Yes ☐ No ☐

72. In the last 3 years, how many such illnesses with (increased) phlegm, did you have which lasted a week or more?

    .... Number of illnesses
    ……………….. No such illnesses
    ……………………. . Does not apply

Past Illnesses

73. Did you have any lung troubles before age of 16? [If participant is over 16 years]

    Yes ☐ No ☐

74. Have you had any of the bronchitis?

    Yes ☐ No ☐ ***(If No Skip, GO to Q75)***

If Yes

1. Was it confirmed by a doctor? Yes ☐ No ☐
2. At what age was the first attack?

   .... years
   ………… does not apply

75. Have you had pneumonia (including bronchopneumonia)

    Yes ☐ No ☐ ***(IF NO skip, GO to Q76)***

If Yes

1. Was it confirmed by a doctor? Yes ☐ No ☐

2. At what age was the first attack?

   .... years
   …………does not apply

76. Have you had chronic bronchitis

Yes ☐ No ☐ ***(If NO skip, GO to Q77)***

If YES

1. Was it confirmed by a doctor? Yes ☐ No ☐
2. Do you still have it? Yes ☐ No ☐ Does not apply ☐
3. At what age was the first attack?

   .... years
   ………… does not apply

77. Have you had asthma?

Yes ☐ No ☐ **(If NO, end of Questionnaire, Thank you the participant)**

If YES

1. Was it confirmed by a doctor? Yes ☐ No ☐
2. Do you still have it? Yes ☐ No ☐ Does not apply ☐
3. At what age was the first attack? .... years…………
4. If you no longer have it, at what age did it stop? ………..years

References

1. How to Create a Random Sample in Excel. Available online: http://www.wikihow.com/Create-a-Random-Sample-in-Excel (accessed on 12 November 2012).

© 2015 by the authors; licensee MDPI, Basel, Switzerland. This article is an open access article distributed under the terms and conditions of the Creative Commons Attribution license (http://creativecommons.org/licenses/by/4.0/).
